# The 2020 WMO Symposium on Climatological, Meteorological and Environmental factors in the COVID-19 pandemic: A special issue from symposium presentations

**DOI:** 10.1016/j.onehlt.2021.100243

**Published:** 2021-04-07

**Authors:** Neville Sweijd, Benjamin F. Zaitchik

**Affiliations:** a Applied Center for Climate and Earth Systems Science, Cape Town, South Africa; b Department of Earth and Planetary Sciences, Johns Hopkins University, Baltimore, MD, USA

**Keywords:** COVID-19, Environmental health, Infectious disease

## Abstract

The COVID-19 pandemic has become one of the great historical events of the modern era, presenting a generational challenge to the world. Questions about the role of weather on SARS-CoV-2 transmission led to the gathering of scientists at an online event, the “International Virtual Symposium on Climatological, Meteorological and Environmental factors in the COVID-19 pandemic,” convened on 4–6 August 2020 under the auspices of the World Meteorological Organization. This collection of papers arise from the Symposium.

The COVID-19 pandemic has become one of the great historical events of the modern era, presenting a generational challenge to the world. The scale and importance of this pandemic, and its potential impact across societies, was recognized early on. Testament to this is that by February 2020 there were already several hundred scientific publications concerning clinical aspects and related matters in the pre-print and peer-reviewed literature. This was the harbinger of a deluge of information that has been generated regarding both the SARS-CoV-2 virus and the disease itself, which has seen what we would argue is one of the broadest mobilizations of the scientific community to focus on a single problem. Researchers from multiple branches of science, engineering, social science, and, of course, medicine, joined the massive response to make a contribution to the global effort to understand, assess, predict, control and obviate the pandemic and its wide-ranging impacts.

Within the domain of natural sciences, population biologists and zoologists, ecological modelers, climate scientists, air quality specialists, geographers and many other disciplines raised considerations that they contended could contribute to grappling with this immediate and existential challenge. Among this scrum of activity, one thread that emerged and became elevated to public discourse by both the media and by officials around the world was the role of weather and climate in the trajectory of the pandemic. An article on the National Public Radio website on the 12th of February 2020 asked “Can Coronavirus Be Crushed By Warmer Weather?” [1] and quoted experts on their speculation that the disease would likely be subject to the same incidence seasonality of similar and common coronavirus illnesses. On March 23rd 2020 the Oxford COVID-19 Evidence Service Team, from the Centre for Evidence-Based Medicine at the University of Oxford, concluded that although evidence was still to be peer-reviewed, “it appears to suggest that weather conditions may influence the transmission of the novel coronavirus (SARS-CoV-2), with cold and dry conditions appearing to boost the spread” [2]. This speculative information was used by world leaders and other officials and this most likely influenced early response policy and strategy. As an example, then United States President Donald Trump, stated on February 10th 2020 that “Now, the virus that we're talking about having to do — you know, a lot of people think that goes away in April with the heat — as the heat comes in. Typically, that will go away in April” [3].

The pandemic initially took hold in the second half of the Northern Hemisphere winter of 2019/2020, spreading from China to Europe and North America in this period. In the Southern Hemisphere, however, the disease manifested in its late summer and autumn, lagged a few months from the initial Northern Hemisphere spread. Given that other coronaviruses and influenza typically exhibit incidence peaks in winter, a fear emerged in the South that the disease itself, and co-morbidity with influenza in the winter season (mid-2020), would be catastrophic and result in a worse situation than the initial outbreak in the northern hemisphere. In the North, it was speculated (and anticipated) that the initial outbreak would be dampened by the effect of the approaching summer season and that that would provide some relief to efforts to combat and prepare for the following winter season. Hence, the questions regarding the role of metrological factors in the epidemiology of COVID-19 became an urgent element of the initial scientific effort.

One immediate scientific challenge for studies of meteorological impacts on SARS-CoV-2 and COVID-19 was that only very short time-series of case counts, hospitalizations and mortalities (some of those of questionable quality) were available. The pressure to complete early analyses in order to quantify the relationships between metrological and climate variability and COVID-19, and establish how important the pending metrological seasonal changes may have been, prompted scientists to substitute space (comparing local epidemic development at a range of geographical scales) for time. This assumed that geographical variability in seasonal progression or variation in prevailing climate in different locales, would drive incidence variability with a discernable signal. This space-for-time approach supported rapid analyses, which began emerging on pre-print servers and journals as early as February 2020. One example is Luo et al. 2020 [4] with a manuscript entitled “The role of absolute humidity on transmission rates of the COVID-19 outbreak” which was posted on MedRixV pre-print server on the 17th of February 2020 (this version has been cited 114 times at the time of writing). Similarly Sajadi et al., 2020, posted a manuscript entitled “Temperature, Humidity and Latitude Analysis to Predict Potential Spread and Seasonality for COVID-19” on the 9th of March 2020, and which was published in the JAMA Network on June 11th 2020 [5]. Various versions of this paper have been cited 387 times at the time of writing. An immediate concern of this inter-geographical approach was how this spatial variation (at ranging scales) would account for regionally varying confounding variables and varying non-pharmaceutical interventions (NPIs). As the pandemic continued over multiple seasons, additional studies attempted to decipher weather and climate signals in longitudinal studies, and these studies also struggled to adjust for the background of rapidly shifting policies and social behaviors that presented time-varying confounders. Hence, the emerging literature was treated with some skepticism.

In order to evaluate the veracity and local applicability if these studies and to further examine this aspects of the pandemic, several groups of scientists coalesced in various parts of the globe. Over the course of the early 2020, some of these groups were formalized. In South Africa, for example, a set of mainly climate scientists set up the COVID-19 Environmental Reference Group which was integrated with a series of scientific investigation and modelling efforts coordinated by the Department of Science and Innovation. In Australia, the Chief Scientist issued a report entitled “Rapid Research Information Forum – Seasonality of COVID-19: Impact on the spread and severity” [6]. To support an international exchange of ideas on this topic, the Group on Earth Observations (GEO)-Health Community of Practice (CoP) convened a global audience for weekly conference calls throughout the early stages of the pandemic. These conversations highlighted shared challenges of short and unreliable COVID-19 records and difficulty in controlling for confounding variables. They also pointed to the potential value to share experiences and research results across climate zones and hemispheres. On the basis of these GEO-Health CoP discussions and a major review paper that took a cross-hemisphere perspective on the pandemic [11], it was decided that an international symposium would be of value. The purpose of the event would be to “elucidate what is known, understood, and can be reliably predicted about environmental variables' influence on the trajectory of the COVID-19 epidemic, from global, hemispheric, regional and local perspectives” [7].

The proposed event was realized as the “International Virtual Symposium on Climatological, Meteorological and Environmental factors in the COVID-19 pandemic,” convened on 4–6 August 2020 under the auspices of the World Meteorological Organization with funding from the U.S. National Oceanographic and Atmospheric Administration (NOAA) and implementation support from the American Geophysical Union (AGU). The symposium included more than 400 participants from 72 countries. The event included invited keynote presentations and panels, breakout discussions, and a scientific virtual poster session, and yielded a summary Outcomes Statement (https://public.wmo.int/en/events/meetings/covid-19-symposium/outcomes), a perspectives piece on the science and communication of studies of meteorological influences on COVID-19 [8], and an entry point for a WMO COVID-19 Task Team, established in parallel to the Symposium organizing effort. In addition, it was agreed to produce a special issue of One Health from contributions to the event, as a record of the technical proceedings. It was thus that this guest editorial team comprising Professor Ben Zaitchik, Dr. Neville Sweijd and Dr. Joy Shumake-Guillemot managed the process of assessing and reviewing contributions that appear in this volume. All contributions to the special issue were passed through rigorous peer review, informed by the research principles outlined in the symposium's Outcomes Statement. We are grateful to the One Health editors and to Elsevier for supporting the production of this issue, and for waiving standard publication fees. We also recognize the contributions of the symposium Scientific Committee (https://public.wmo.int/en/events/meetings/covid-19-symposium/scientific-committee) in planning and presenting the symposium, and the peer reviewers for the issue, who contributed their time and expertise during an extremely demanding period.

The outcome statement of the symposium recognized that the study of the role of climate and metrological factors in the epidemiology of COVID-19 would remain very challenging. This was emphasized in subsequent comment [9] and is now the subject of a report by the WMO COVID-19 Task Team [10].

Since the symposium, which was held in July 2020, a lot of water has passed under the bridge, with second and third waves of incidence peaks occurring in many regions of the world, seemingly independent of metrological seasonal changes (but perhaps not their behavioral influences). Added to this has been the more recent introduction of vaccine strategies which has added additional variables in terms of the efficacy of the range of options available together and the varying local strategies and success of their rollout. To further muddy the waters, new variants of SARS-CoV-2, with varying epidemiological properties and vaccine efficacy, have emerged and spread amorphously across countries.

The work in this volume is thus an important record of early thinking and presents some significant contributions to this aspect of the pandemic which remains relevant, and most likely will continue to be in the coming seasons and years. It also represents a small sample of the unprecedented and laudable effort that scientists across the globe undertook to contribute to this specific aspect of the pandemic and we are grateful to the many colleagues who did and who continue to conduct research into this question.
